# Abemaciclib-induced myocarditis in early breast cancer: a case report

**DOI:** 10.1186/s40959-025-00442-2

**Published:** 2026-01-14

**Authors:** Alexander JP Fulton, Mun Cheang, Maria TA Wetscherek, Paul Cacciottolo, Jean E Abraham

**Affiliations:** 1 Cambridge University NHS Foundation Trust, Cambridge, UK; 2 Precision Breast Cancer Institute, Department of Oncology, University of Cambridge, Cambridge, UK; 3 Royal Papworth Hospital, Cambridge, UK; 4 Department of Radiology, University of Cambridge, Cambridge, UK

**Keywords:** Breast cancer, Abemaciclib, Myocarditis

## Abstract

**Background:**

Abemaciclib, a selective cyclin-dependent kinase 4 and 6 (CDK4/6) inhibitor, is standard adjuvant therapy for hormone receptor (HR)-positive, HER2-negative early breast cancer. While gastrointestinal and haematologic toxicities are well recognised, cardiotoxicity—particularly myocarditis—is extremely rare. To our knowledge, myocarditis has not previously been reported in early breast cancer. This case describes a case of acute abemaciclib-induced myocarditis and explores a potential genetic predisposition involving an *RHBDF2* germline variant affecting growth factor signalling pathways.

**Case presentation:**

A 39-year-old female with high-risk HR-positive, HER2-negative early breast cancer commenced adjuvant abemaciclib with letrozole following completion of chemotherapy and radiotherapy. Within 24 h, she developed acute chest pain with an elevated troponin I (8457 ng/L), new T-wave inversion on her ECG, and a reduced left ventricular ejection fraction (47%) on echocardiography. CT imaging excluded coronary or aortic pathology. Cardiac magnetic resonance imaging demonstrated mid-inferoseptal late gadolinium enhancement consistent with acute myocarditis. Abemaciclib was immediately discontinued, and bisoprolol and ramipril were initiated. Her symptoms and cardiac function improved rapidly, with normalisation of left ventricular ejection fraction (68%). Genetic testing identified a germline RHBDF2 missense variant, which is predicted to alter tumour necrosis factor alpha and epidermal growth factor signalling, potentially predisposing to cardiomyocyte injury based on published in vitro evidence.

**Conclusions:**

To our knowledge, this is among the earliest reported cases of abemaciclib-induced myocarditis in early breast cancer. Awareness of this potential toxicity is crucial, as early recognition and management is pivotal to prevent lasting cardiac dysfunction. Genetic variants influencing inflammatory or growth factor pathways may contribute to individual susceptibility, warranting further investigation. This case highlights the importance of combined cardio-oncology care in the early recognition of cardiotoxicity when introducing CDK4/6 inhibitors.

## Introduction

Targeting of cyclin-dependent kinases 4 and 6 (CDK4/6) has improved clinical outcomes in hormone receptor (HR) positive breast cancer. Abemaciclib, a selective CDK 4/6 inhibitor, is used as the standard of care adjuvant treatment of high-risk HR-positive, HER2-negative early breast cancer. While commonly associated with gastrointestinal and hematologic toxicities, cardiotoxicity, particularly myocarditis, is a rare but potentially life-threatening adverse effect. To date, cardiotoxicity from CDK4/6 inhibitors is infrequently reported and as such is rarely listed as a potential toxicity [1]. Here we describe a patient with early breast cancer that developed acute myocarditis after commencing adjuvant abemaciclib.

### Clinical background

A 39-year-old female with a history of early breast cancer (T2 N3 M0, grade 3 invasive carcinoma, HR-positive, HER2-negative) was initiated on adjuvant letrozole (2.5 mg once daily) in February 2024 and abemaciclib (150 mg twice daily) on 22nd April 2024. She had recently completed adjuvant paclitaxel, epirubicin and cyclophosphamide chemotherapy followed by 40 Gy in 15 fractions of right chest wall and right axilla radiotherapy. She had no anthracycline exposure prior to her breast cancer diagnosis. As such, her total epirubicin exposure was 360mg/m^2^ between 19th December 2023 and 30th January 2024.

Her past medical history included a bicuspid aortic valve and an aneurysmal atrial septum. She had no known history of coronary artery disease. Her uncle had hypertrophic cardiomyopathy but she had no personal or family history of myocardial infarction. Baseline echocardiography at the time of diagnosis demonstrated normal cardiac function with no regional wall motion abnormalities. She notably had a germline missense variant in *RHBDF2*, a gene that plays a role in the modulation of growth factor signalling such as epidermal growth factor (EGF) and tumour necrosis factor alpha (TNFα) [2, 3]. This variant had not previously been reported but was predicted to have a functional consequence using Polyphen modelling.

### Clinical course

One day after starting abemaciclib, she presented to the emergency department with gradual onset heavy central chest pain radiating into her left arm and associated with intermittent palpitations. Her symptoms then eased over 1.5 h. On examination, her vital signs were within normal limits: BP 114/65 mmHg, HR 76 bpm, RR 14/min, temperature 36.5 °C, and oxygen saturation 100%. Cardiac auscultation revealed an ejection systolic murmur. Her respiratory examination was unremarkable, and she had no notable peripheral oedema.

She had no preceding viral symptoms and had not experienced similar symptoms previously.

### Diagnostic workup

**Fig. 1:**
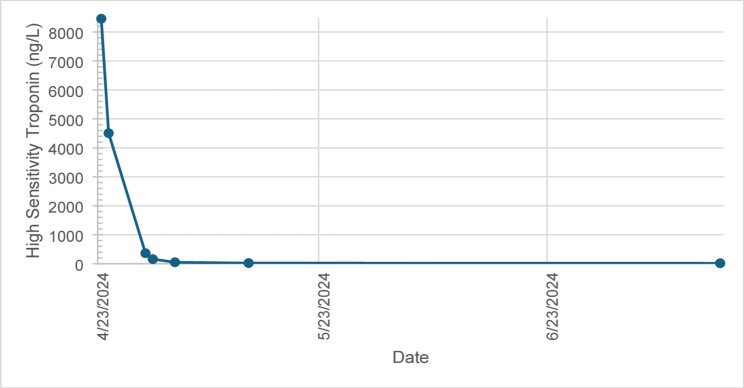
Serial high sensitivity troponin levels demonstrate a raised level at the onset of chest pain, followed by normalisation after discontinuation of abemaciclib on 24/04/2024

**Fig. 2:**
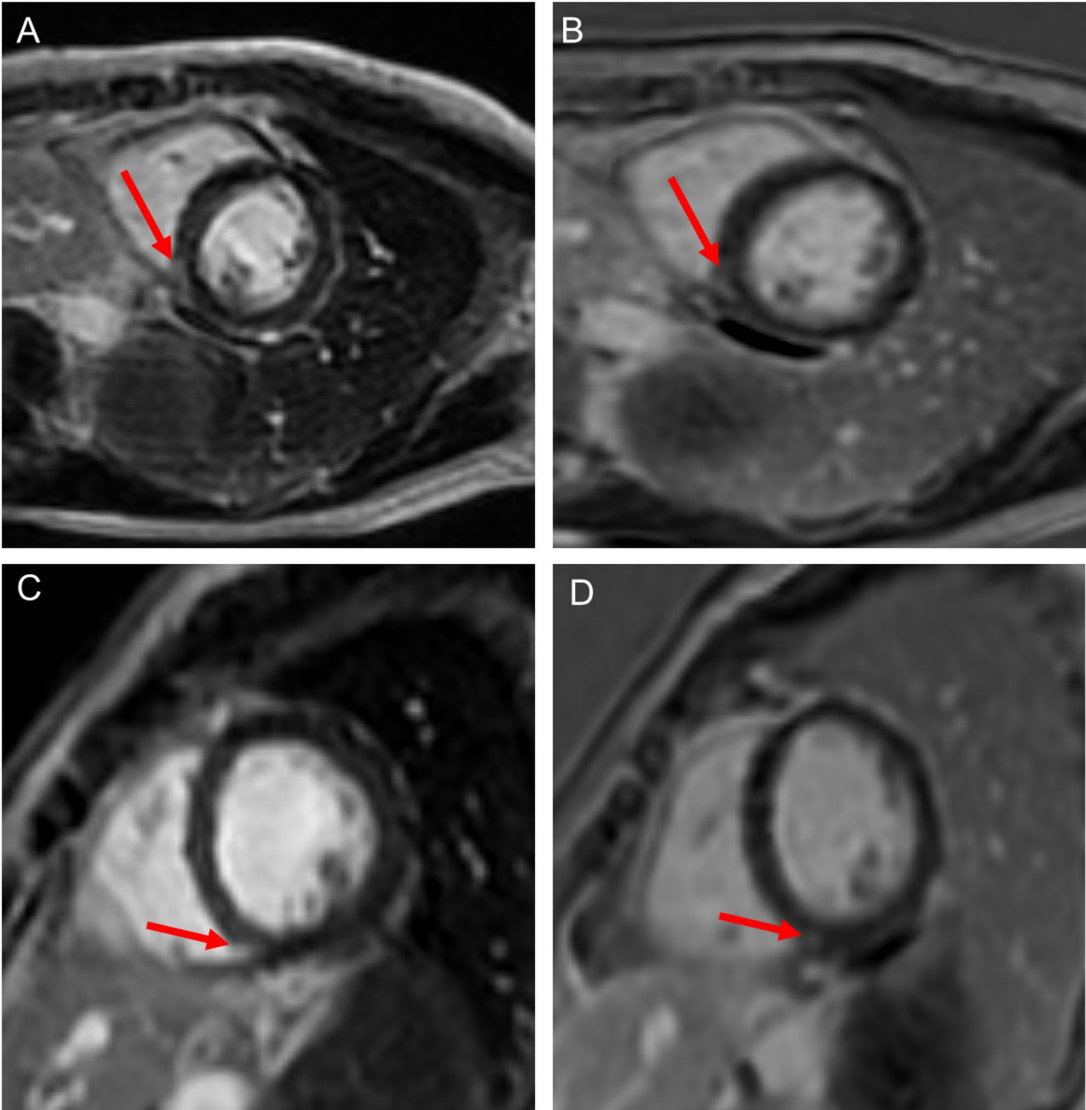
Cardiac magnetic resonance (CMR) imaging at baseline (**A**, **B**) and 3-month follow-up (**C**, **D**). (**A**) Late gadolinium enhancement (LGE) short-axis (SAX) image of the mid-inferoseptum. (**B**) Single-shot LGE SAX of the mid-inferoseptum. (**C**) LGE SAX at follow-up. (**D**) Single-shot LGE SAX at follow-up

Initial laboratory investigations demonstrated a normal white cell count and C reactive protein but an elevated troponin I level at 8457 ng/L (normal < 39 ng/L), indicating myocardial injury. An electrocardiogram (ECG) demonstrated normal sinus rhythm with new T wave inversion in leads V5, V6, II, III, AVF. Transthoracic echocardiogram (TTE) imaging revealed a left ventricular ejection fraction (LVEF) of 47% (reduced from a baseline of 61%, GLS − 20%) with basal, midinferior and inferolateral wall hypokinesis. CT aorta imaging with contrast demonstrated no acute aortic syndrome, pulmonary emboli or evidence of significant coronary artery plaque or stenosis. Serial troponin I levels rapidly reduced until normalisation post abemaciclib discontinuation (Fig. 1). Subsequent cardiac magnetic resonance imaging (CMR) demonstrated an improved systolic function (LVEF 64%) with evidence of late gadolinium enhancement in the mid-inferoseptum, consistent with resolving acute myocarditis (Fig. 2). Given the clinical presentation, imaging findings, and recent initiation of abemaciclib, a diagnosis of abemaciclib-induced acute myocarditis was made.

### Management

Abemaciclib was immediately discontinued and cardioprotective bisoprolol (1.25 mg daily) and ramipril (1.25 mg daily) were commenced. She was admitted to the inpatient cardiology unit after her initial high sensitivity troponin I was raised.

Cardiac telemetry monitoring demonstrated no evidence of significant arrhythmia.

Following exclusion of acute coronary or aortic syndromes, she was diagnosed with acute myocarditis associated with transient LV dysfunction and she received standard of care cardioprotective management. She was discharged home on day 10 of admission after the CMR demonstrated a resolving myocarditis, along with no recurrence of her symptoms and a normalising high sensitivity troponin I.

She subsequently restarted letrozole on 30/4/24 and discontinued abemaciclib indefinitely.

### Follow-up and outcome

Repeat CMR imaging on 11th August 2024, after four months of bisoprolol and ramipril therapy, showed an improvement in LVEF to 68% and almost complete resolution of subepicardial LGE (Fig. 2). Her bisoprolol and ramipril are ongoing as cardioprotective treatment. She continued letrozole and did not restart abemaciclib. To date, she has no evidence of breast cancer recurrence.

## Discussion

We report a case of acute myocarditis occurring within 24 h of initiating abemaciclib. Prior to this treatment, she had normal cardiac function and no identifiable risk factors for acute cardiac dysfunction. Following discontinuation of abemaciclib and commencement of cardioprotective therapy with bisoprolol and ramipril, her cardiac function improved and ultimately returned to baseline. Importantly, no changes or persistent reductions in cardiac function were observed during prior letrozole monotherapy.

Abemaciclib is a selective inhibitor of cyclin‑dependent kinases 4 and 6 (CDK4/6). By blocking phosphorylation of the retinoblastoma protein, it prevents cell cycle progression from G1 to S phase, resulting in cell cycle arrest, inhibition of tumour proliferation, and ultimately cell death. CDK4/6 inhibitors in combination with endocrine therapy are licensed for hormone receptor‑positive, HER2‑negative breast cancer. Abemaciclib, when used alongside endocrine therapy, has become the standard adjuvant treatment for patients at high risk of recurrence. This includes those with ≥ 4 positive lymph nodes or 1–3 positive nodes with either grade 3 disease or tumour size ≥ 50 mm. The addition of abemaciclib significantly improved invasive disease‑free survival at 48 months from 78.6% to 85.5% when compared with endocrine therapy alone [4].

CDK4/6 inhibitor toxicity and discontinuation are often associated with diarrhoea, myelosuppression, liver dysfunction and fatigue [4]. Cardiac toxicity has been previously reported as a ‘rare’ toxicity from abemaciclib and, in the metastatic setting, heart failure/cardiomyopathy have been observed in 5.8% of patients receiving this treatment [5]. However, abemaciclib associated acute myocarditis, to our knowledge, has not been previously reported in early breast cancer [1]. It is therefore important to highlight this potentially long term detrimental cardiovascular toxicity as an associated risk of abemaciclib treatment.

The exact underlying mechanism of abemaciclib induced myocarditis is unclear. Preclinical studies have suggested CDK4/6 inhibition leads to alteration in potassium and sodium channel activity, vascular inflammation and left ventricular remodelling [5]. Recent preclinical data suggests that CDK4/6 inhibition leads to cardiomyocyte apoptosis secondary to activation of the Hippo signalling pathway [6]. In this context, one possible explanation for the cardiomyocyte toxicity observed in this patient is the presence of a germline *RHBDF2* missense variant. This could influence growth factor signalling pathways, including TNFα signalling. Enhanced TNFα signalling has been reported to interact with and potentially amplify Hippo pathway activity [7]. It is possible that this could have contributed to the patient’s presentation, however, this remains a speculative hypothesis based on limited evidence and would require dedicated mechanistic studies to explore further. In our case, 24–48 h of exposure to abemaciclib led to acute myocardial dysfunction, perhaps implicating a direct cardiomyocyte toxicity of abemaciclib. Early recognition and discontinuation enabled recovery in cardiac function within days. It is unclear, however, if more prolonged treatment may have led to irreversible reduction in cardiac function in an individual without prior cardiac risk factors.

## Conclusion

This case highlights a serious complication of abemaciclib therapy that may not be as rare as previously thought. Clinicians should be aware of the potential for abemaciclib-induced myocarditis and consider it in the differential diagnosis when patients present with new cardiac symptoms. Prompt intervention and supportive care can result in favourable outcomes within days of commencing abemaciclib, though the long-term effects of more extended use remain unclear. Multidisciplinary oncology and cardiology input is essential, and CMR imaging may assist in identifying myocarditis as the underlying cause. Further research into the mechanism of CDK4/6 inhibitor induced cardiomyocyte injury is warranted. This would enable identification of individuals particularly at risk of cardiotoxicity with abemaciclib and perhaps a subsequent tailored approach to their treatment.

### Patient perspective

As a patient facing cancer treatment, knowing both the benefits and potential risks of therapy is deeply important. Experiencing unexpected side effects, like heart inflammation from abemaciclib, can be frightening and overwhelming. Greater understanding and reporting of these rare toxicities are vital—not only so doctors can monitor and manage them promptly, but also so patients can make truly informed decisions about their care. I hope that sharing my experience can help future patients feel safer and better prepared as they navigate their treatment journey.

## Data Availability

No datasets were generated or analysed during the current study.

## Abbreviations

CDK4/6: Cyclin Dependant Kinase 4 and 6
HR: Hormone Receptor
HER2: Human Epidermal Growth Factor Receptor 2
RHBDF2 gene: Rhomboid 5 Homolog 2 gene
EGF: Epidermal Growth Factor
TNFα: Tumour Necrosis Factor Alpha
ECG: Electrocardiogram
TTE: Transthoracic Echocardiogram
LVEF: Left Ventricular Ejection Fraction
GLS: Global Longitudinal Strain
CMR: Cardiac Magnetic Resonance
MRI: Magnetic Resonance Imaging
LGE: Late Gadolinium Enhancement

## References

[1] Kim JH. Profiling the cardiovascular toxicities of CDK4/6 inhibitors: A Real-World pharmacovigilance study. Cancers (Basel). 2024;16(16):2869. 39199640 10.3390/cancers16162869 PMC11352810

[2] Adrain C, Zettl M, Christova Y, Taylor N, Freeman M. Tumor necrosis factor signaling requires iRhom2 to promote trafficking and activation of TACE. Science. 2012;335(6065):225–8. 22246777 10.1126/science.1214400 PMC3272371

[3] Hosur V, Johnson KR, Burzenski LM, Stearns TM, Maser RS, Shultz LD. Rhbdf2 mutations increase its protein stability and drive EGFR hyperactivation through enhanced secretion of Amphiregulin. Proc Natl Acad Sci U S A. 2014;111(21):E2200–9. 24825892 10.1073/pnas.1323908111 PMC4040562

[4] Johnston SRD, Toi M, O’Shaughnessy J, Rastogi P, Campone M, Neven P, et al. Abemaciclib plus endocrine therapy for hormone receptor-positive, HER2-negative, node-positive, high-risk early breast cancer (monarchE): results from a Preplanned interim analysis of a randomised, open-label, phase 3 trial. Lancet Oncol. 2023;24(1):77–90. 36493792 10.1016/S1470-2045(22)00694-5 PMC11200328

[5] Fradley MG, Nguyen NHK, Madnick D, Chen Y, DeMichele A, Makhlin I, et al. Adverse cardiovascular events associated with Cyclin-Dependent kinase 4/6 inhibitors in patients with metastatic breast cancer. J Am Heart Assoc. 2023;12(12):e029361. 37301767 10.1161/JAHA.123.029361 PMC10356048

[6] Zhou Y, Li Y, Shen J, Li J, Li X. Abemaciclib induces apoptosis in cardiomyocytes by activating the Hippo signaling pathway. Acta Biochim Biophys Sin (Shanghai). 2020;52(8):875–82. 32556311 10.1093/abbs/gmaa066

[7] Oceandy D, Amanda B, Ashari FY, Faizah Z, Azis MA, Stafford N. The Cross-Talk between the TNF-α and RASSF-Hippo signalling pathways. Int J Mol Sci. 2019;20(9):2346. 10.3390/ijms20092346 PMC6539482 31083564

